# An integrated yeast‐based process for *cis*,*cis*‐muconic acid production

**DOI:** 10.1002/bit.27992

**Published:** 2021-11-24

**Authors:** Guokun Wang, Aline Tavares, Simone Schmitz, Lucas França, Hugo Almeida, João Cavalheiro, Ana Carolas, Süleyman Øzmerih, Lars M. Blank, Bruno S. Ferreira, Irina Borodina

**Affiliations:** ^1^ The Novo Nordisk Foundation Center for Biosustainability Technical University of Denmark Lyngby Denmark; ^2^ Tianjin Institute of Industrial Biotechnology Chinese Academy of Sciences Tianjin China; ^3^ Biotrend – Inovação e Engenharia em Biotecnologia SA Cantanhede Portugal; ^4^ Institute of Applied Microbiology‐iAMB, Aachen Biology and Biotechnology‐ABBt RWTH Aachen University Aachen Germany

**Keywords:** fermentation, metabolic engineering, purification, recovery, *Saccharomyces cerevisiae*

## Abstract

*Cis,cis‐muconic* acid (CCM) is a promising polymer building block. CCM can be made by whole‐cell bioconversion of lignin hydrolysates or *de novo* biosynthesis from sugar feedstocks using engineered microorganisms. At present, however, there is no established process for large‐scale CCM production. In this study, we developed an integrated process for manufacturing CCM from glucose by yeast fermentation. We systematically engineered the CCM‐producing *Saccharomyces cerevisiae* strain by rewiring the shikimate pathway flux and enhancing phosphoenolpyruvate supply. The engineered strain ST10209 accumulated less biomass but produced 1.4 g/L CCM (70 mg CCM per g glucose) in microplate assay, 71% more than the previously engineered strain ST8943. The strain ST10209 produced 22.5 g/L CCM in a 2 L fermenter with a productivity of 0.19 g/L/h, compared to 0.14 g/L/h achieved by ST8943 in our previous report under the same fermentation conditions. The fermentation process was demonstrated at pilot scale in 10 and 50 L steel tanks. In 10 L fermenter, ST10209 produced 20.8 g/L CCM with a CCM yield of 0.1 g/g glucose and a productivity of 0.21 g/L/h, representing the highest to‐date CCM yield and productivity. We developed a CCM recovery and purification process by treating the fermentation broth with activated carbon at low pH and low temperature, achieving an overall CCM recovery yield of 66.3% and 95.4% purity. In summary, we report an integrated CCM production process employing engineered *S. cerevisiae* yeast.

## Graphical abstract

An integrated yeast‐based process for cis, cis‐muconic acid (CCM) production was described. Wang and co‐workers reprogrammed the CCM‐producing yeast S. cerevisiae strain by increasing PEP supply and rewiring the shikimate pathway, established a CCM bioproduction process that was robust in the scale‐up, and developed the first CCM recovery and purification process for yeasts.

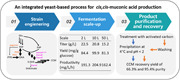

## 1 INTRODUCTION

Muconic acid (2,4‐hexadienedioic acid) is a six‐carbon dicarboxylic acid with two conjugated double bonds. It occurs in three isomeric forms: *cis,cis*‐muconic acid (CCM), *trans,trans*‐muconic acid, and *cis,trans*‐muconic acid. Muconic acid is a promising platform chemical. It can be used as the bio‐based starting material for making common polymer precursors (Averesch & Kromer, 2018; Khalil et al., 2020; Matthiesen et al., 2016), such as 3‐hexenedioic acid, adipic acid, and terephthalic acid (TPA), which are otherwise manufactured from fossil resources.

Muconic acid can be produced by chemical synthesis, bioconversion of lignin, or microbial fermentation of sugars. Chemical synthesis routes use nonrenewable petroleum‐derived chemicals as feedstocks: diethyl 2,3‐dibromoadipate, dimethyl 3,4‐dibromohexanedioate, phenol, *o*‐coumaric acid, and others. The yields range between 4% and 60% (Khalil et al., 2020). For instance, oxidation of phenol for 10 days or *o*‐coumaric acid for 14 days using peracetic acid generates CCM at a molar yield of 35% and 4%, respectively (Böeseken, 1932; Khalil et al., 2020). Some microbial strains, such as *Pseudomonas putida* KT2440, *Corynebacterium glutamicum*, and *Amycolatopsis* sp. ATCC 39116 can degrade lignin‐based aromatic compounds (Barton et al., 2018; Becker et al., 2018; Vardon et al., 2015), such as catechol, phenol, guaiacol, and *p*‐coumaric acid, with CCM as the degradation product. Microbes overexpressing catechol 1,2‐dioxygenase, such as *P. putida* KT2440 (Kohlstedt et al., 2018), *C. glutamicum* (Becker et al., 2018), and *Escherichia coli* (Kaneko et al., 2011), can convert catechol to CCM with a nearly 100% yield, with the highest titer of 85 g/L achieved in *C. glutamicum* (Becker et al., 2018). *P. putida* KT2440 strain, engineered with phenol hydroxylase and catechol‐induced catechol 1,2‐dioxygenase, produced 13 g/L CCM from the hydrothermally depolymerized softwood lignin (Kohlstedt et al., 2018). In these bioconversion processes, glucose or another fermentable carbon source must be added to support cellular growth and energy generation.

**Figure 1:**
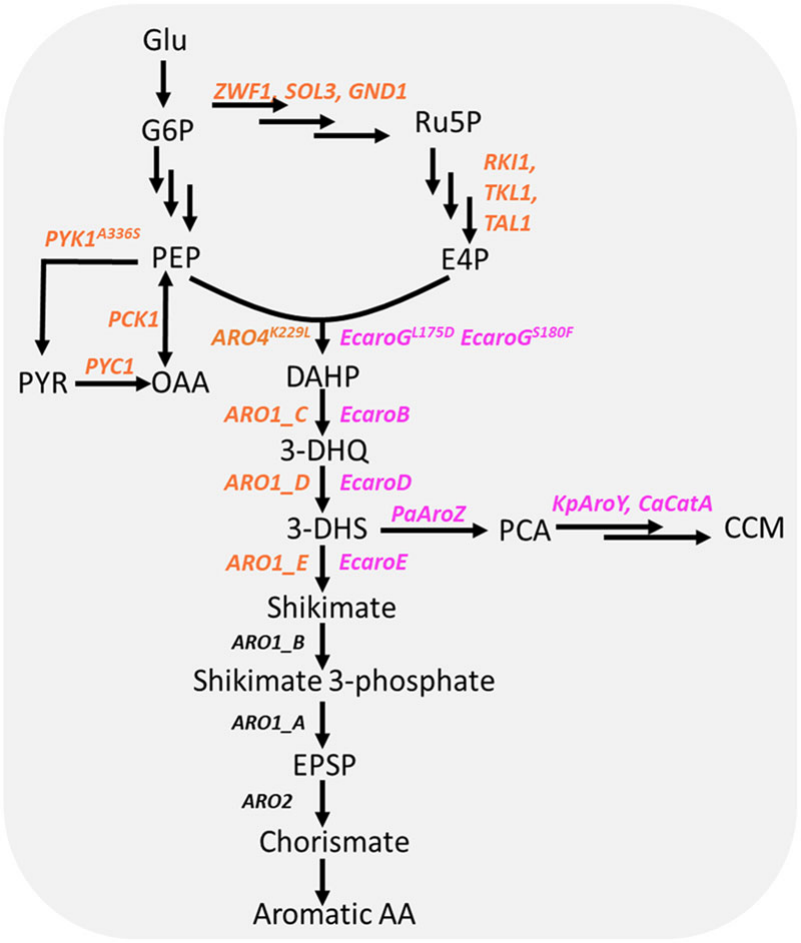
Biosynthetic pathway for *cis*,*cis*‐muconic acid production in *Saccharomyces cerevisiae*. Native and heterologous enzymes manipulated in this study are marked in orange and purple, respectively. EcaroG: DAHP synthase from *Escherichia coli*, EcaroB: 3‐dehydroquinate synthase from *E. coli*, EcaroD: 3‐dehydroquinase from *E. coli*, and EcaroE: shikimate dehydrogenase from *E. coli*, PaAroZ: DHS dehydratase from *Podospora anserina*, KpAroY: subunits of functional PCA decarboxylase from *Klebsiella pneumoniae*, CaCatA: catechol 1,2‐dioxygenase from *Candida albicans*; Glu: glucose, G6P: glucose 6‐phosphate, Ru5P: ribulose 5‐phosphate, PEP: phosphoenolpyruvate, E4P: erythrose 4‐phosphate, PYR: pyruvate, OAA: oxaloacetic acid, DAHP: 3‐deoxyarabinoheptulosonate 7‐phosphate, 3‐DHS: 3‐dehydroshikimate, PCA: protocatechuic acid, CCM: *cis*,*cis*‐muconic acid, 3‐DHQ: 3‐dehydroquinate, EPSP: 5‐enolpyruvylshikimate‐3‐phosphate, AA: amino acid

It is also possible to engineer microbial strains to biosynthesize CCM *de novo* from sugars. In the cells, sugars are catabolized through glycolytic (Embden‐Meyerhof‐Parnas and/or Entner–Doudoroff pathways) and pentose phosphate pathways, generating phosphoenolpyruvate (PEP) and erythrose 4‐phosphate (E4P). PEP and E4P are condensed to 3‐dehydroshikimate (3‐DHS) by the sequential action of 3‐deoxy‐d‐arabino‐heptulosonate‐7‐phosphate (DAHP) synthase, 3‐dehydroquinate (3‐DHQ) synthase, and 3‐dehydroquinase. 3‐DHS is then converted to protocatechuic acid (PCA), catechol, and CCM, by correspondingly DHS dehydratase, PCA decarboxylase, and catechol 1,2‐dioxygenase (Figure 1). Model chassis organisms, *E. coli* and *Saccharomyces cerevisiae*, do not have the last three enzymatic activities and require genetic engineering. The *E. coli* strain engineered for enhanced 3‐DHS supply and expressing the 3‐DHS‐to‐CCM pathway produced up to 59 g/L of CCM from glucose, with a yield of 0.3 g/g glucose and a productivity of 0.9 g/L/h (Bui et al., 2014). The CCM titers reported in yeasts are lower than in *E. coli*, but yeast fermentation presents several advantages that make it attractive to develop a yeast‐based process. Yeast fermentation can be carried out at acidic rather than neutral pH, which reduces the risk of contamination, and yeasts are phage‐resistant. The CCM production in yeasts was improved in different studies by optimizing the 3‐DHS‐to‐CCM pathway (Skjoedt et al., 2016; Weber et al., 2017; Wang et al., 2020), boosting 3‐DHS supply from PEP and E4P (Bruckner et al., 2018; Curran et al., 2013; Leavitt et al., 2017), tailoring the pentose phosphate pathway (Curran et al., 2013; Suástegui et al., 2017), biosensor‐aided genome engineering (Leavitt et al., 2017; Snoek et al., 2018; Wang et al., 2020), transporter engineering (Wang et al., 2021), and by fermentation optimization (Pyne et al., 2018; Wang et al., 2020). The highest CCM titer (20.8 g/L) was achieved in a CEN.PK background strain with a yield of 66.3 mg/g glucose and productivity of 139 mg/L/h, as reported in our previous publication (Wang et al., 2020). The titer, productivity, and yield of the yeast cell factory still need further improvement to achieve a commercially viable CCM production. Moreover, the engineered strains should be tested at a pilot scale to evaluate the strains' robustness and validate the absence of scale‐dependent discrepancies.

CCM recovery and purification methods have been reported for *P. putida* fermentation (Kohlstedt et al., 2018; Vardon et al., 2015), where the substrates for the bioconversion were catechol (Kohlstedt et al., 2018) and *p*‐coumaric acid (Vardon et al., 2015). The process involved: (i) activated carbon treatment removing the color and nontarget aromatic PCA (Kohlstedt et al., 2018; Vardon et al., 2015), (ii) CCM precipitation under low pH (≤2; Kohlstedt et al., 2018; Vardon et al., 2015) and low temperature (5°C; Vardon et al., 2015), and (iii) spray drying (Kohlstedt et al., 2018). The resulting CCM was >97% pure, and the overall recovery yield was 74% (Vardon et al., 2015). No recovery or purification process has been reported for yeast‐based CCM production. Further, little attention has been given to the influence of the purification conditions on the isomeric purity of CCM.

In this study, we combined yeast strain engineering, controlled fed‐batch fermentation and scale‐up, and CCM recovery and purification, to establish an integrated process for CCM production by yeast fermentation.

## 2 MATERIALS AND METHODS

### 2.1 Strain construction

*E. coli* DH5α was used for plasmid construction and propagation. Yeast strains (Table S1) used in this study were all derived from CEN.PK113‐7D (Entian & Kotter, 2007). The yeast strains were all constructed using a Cas9‐assisted approach (Jessop‐Fabre et al., 2016). Guide RNA plasmids for a single target (Table S2) were constructed using Gibson assembly with Gibson Assembly® Master Mix (New England BioLabs), with the 20 bp gRNA as the homology region. Double gRNA plasmids (Table S2) were constructed using USER cloning (Jensen et al., 2014). Plasmids for *PYK1* mutations that consist of gRNA and donor fragments were constructed using Gibson assembly with the adaptors of synthesized DNA fragments (Table S3, by Twist Bioscience) as a homology region.

DNA fragments for genome integration were constructed by overlap PCR (Zhou et al., 2012; Table S4) or by NotI digestion of plasmids constructed using the EasyClone method (Jensen et al., 2014). In generating yeast strains overexpressing three pentose pathway genes, three DNA fragments that carry *CYC1* and *ADH1* terminators at the end as homology arms, were employed.

Yeast transformation was carried out using the standard lithium acetate method (Gietz & Woods, 2002). Upon heat shock, transformants were recovered in synthetic defined (SD) complete medium at 30°C for 2 h and then plated onto selective plates. Transformants for genome integration were verified by genomic PCR validation. Transformants for *PYK1* mutation were validated via the sequencing of the genomic PCR product.

### 2.2 Medium and strain cultivation

*E. coli* strains were cultivated on Luria–Bertani (LB) medium containing 100 mg/L ampicillin at 37°C. Yeast strains were maintained on YPD (10 g/L yeast extract, 20 g/L peptone, 20 g/L glucose). Yeast transformants were selected on YPD with 100 mg/L nourseothricin or SD‐Ura (20 g/L glucose, 6.7 g/L yeast nitrogen base, complete supplement mixture lacking uracil) plates. Solid plates contained 20 g/L agar.

Liquid media, including YPD, mineral/DELFT medium (pH 6.0; Jensen et al., 2014), and Feed‐In Time (FIT) medium (mineral medium where 20 g/L glucose is substituted with 60 g/L Enpresso® EnPump 200 substrate and 0.3% [v/v] Enpresso® Reagent A) were employed for yeast cell cultivation. Uracil was supplemented in a final concentration of 50 mg/L for strains lacking *URA3*.

Yeast cell cultivation in liquid medium was performed at 30°C throughout this study, in 96‐deep well plate at 300 rpm in a New Brunswick™ Innova® 44 shaker. Fresh single colonies were first pre‐cultured in 500 μl YPD or mineral medium overnight, and the resulting pre‐culture was sub‐inoculated into 600 μl mineral medium for sub‐cultivation. The mineral medium was preferentially used for pre‐culture, whereas YPD was used when the strains that have growth defects on the mineral medium were included. For the same batch cultivation, either mineral medium or YPD was used for the pre‐culture of all tested strains.

### 2.3 Optical density measurement

Optical density at 600 nm (OD_600_) was measured using a microtiter plate reader BioTek Synergy MX (BioTek). A volume of 200 µl yeast culture in an appropriate dilution (1–10×) was tested with the diluted medium as a blank.

### 2.4 Metabolite quantification

The CCM and PCA concentrations were analyzed using HPLC as previously described (Wang et al., 2020). Briefly, the 72 h subcultures were diluted 10 or 20 times with water, and the supernatant was analyzed using HPLC with Aminex HPX‐87H ion exclusion column kept at 60°C. CCM and PCA were detected at UV 250 and 220 nm, respectively.

### 2.5 Fed‐batch fermentation

Lab‐scale fed‐batch fermentations were carried out in 2 L working volume controlled bioreactors (BioFlo115 from New Brunswick Scientific/Eppendorf) at a controlled temperature of 30°C using an electrical heat blanket and an internal cooling coil. Pilot‐scale fermentations were carried out in stainless steel, in situ sterilized fermenters with 10 and 50 L working volume fermenters (BioFlo415 and BioFlo610, respectively, from New Brunswick Scientific/Eppendorf), at a controlled temperature of 30°C using an external jacket through which thermal fluid (cold water or steam) was circulated. The medium for the starting batch fermentation stage (1.3 L in the 2 L fermenters and 6.0 L in the 10 L fermenter) contained 10 g/L yeast extract, 10 g/L (NH_4_)_2_SO_4_, 4 g/L KH_2_PO_4_, 0.2 g/L NaCl, 1.5 g/L MgSO_4_.7H_2_O, 0.265 g/L CaCl_2_·2H_2_O and 10 mL/L of a trace metals solution (comprised of 1 g/L Na_2_EDTA, 0.2 g/L ZnSO_4_·7H_2_O, 0.1 g/L CaCl_2_·2H_2_O, 0.5 g/L FeSO_4_·7H_2_O, 0.02 g/L Na_2_MoO_4_·2H_2_O, 0.02 g/L CuSO_4_·5H_2_O, 0.7 g/L Co(NO_3_)_2_·6H_2_O, 0.13 g/L MnSO_4_·H_2_O and 9.1 g/L MgSO_4_·7H_2_O). Overnight cultures of ST10209 in 125 ml YPD medium in a 500‐ml shake flask were used for the inoculation (one for the 2 L fermenter and four for the 10 L fermenter), while five 2000 ml flasks with 500 ml YPD medium each were used to inoculate the 50 L fermenter. A feed solution containing 600 g/L glucose, 45 g/L KH_2_PO_4_, 24 g/L MgSO_4_, 30 g/L (NH_4_)_2_SO_4_, 1.2 g/L CaCl_2_, and 150 mL/L of trace metals solution. Filter‐sterilized air was fed at 1 vvm through an air sparger. The dissolved oxygen was measured using a polarographic electrode and controlled automatically at 20% saturation by adjusting the stirring speed. The 2 L bioreactors were equipped with two 6‐blade Rushton turbines and four baffles, while the 10 and 50 L bioreactors had three 6‐blade Rushton turbines. The pH was controlled at 6.0 ± 0.1 by the automatic addition of H_2_SO_4_ 2 N or NH_4_OH 14%. A foam sensor was installed and antifoam (Simethicone 30%, Dow Corning) added automatically whenever foam was detected. An external pump (Multiflow from Lambda Instruments for the 2 L fermenters and Watson Marlow 120 U for the 10 and 50 L fermenters) was used to add the feed solution. Online data acquisition and control was performed through dedicated software (Biocommand from New Brunswick Scientific/Eppendorf) which was interfaced with the feeding pump. An automatic sampler was used to collect frequent samples from the 2 L fermentations, while the samples from the 10 and 50 L fermentations were collected manually.

### 2.6 CCM recovery and purification

The fermentation broth was centrifuged at 13,881 rcf (8000 rpm – Sorvall Lynx 6000 centrifuge) and 20°C for 20 min. The pellet was discarded, and the supernatant was incubated at 35°C and 150 rpm for 60 min in an orbital shaker (New Brunswick Scientific, Inova 43) in the presence of 10% (w/w) of activated carbon (Sigma‐Aldrich, >90%). The activated carbon and the adsorbed impurities were separated from the liquid fraction by centrifugation at 13,881 rcf (8000 rpm – Sorvall Lynx 6000 centrifuge) and 25°C for 10 min and the supernatant was processed by vacuum filtration using a 0.45 µm nylon filter (Whatman) to obtain a particle‐free filtrate. The filtrate was cooled below 4°C using a water/ice bath and the pH was adjusted below 2 using H_2_SO_4_ (95%–97%), causing the precipitation of the CCM in solution. The suspension was incubated for 1 h at 4°C to maximize precipitation. The precipitated CCM was transferred to a Büchner funnel, and the supernatant was removed by dead‐end vacuum filtration, using a 0.45 µm nylon filter (Whatman). The supernatant was discarded, and the filter containing the isolated CCM was dried in the oven for 48 h at 30°C.

The used activated carbon was re‐suspended in reverse osmosis water and centrifuged at 13,881 rcf (8000 rpm – Sorvall Lynx 6000 centrifuge) and 25°C for 10 min to promote the desorption of CCM that may have been retained. The liquid fraction was collected and processed as above to recover dry CCM crystals. The CCM recovery from the activated carbon protocol was repeated to increase the recovery yield and assess the impact on the final purity. After drying, the recovered CCM precipitate was ground and homogenized in a mortar. The recovery yield was calculated gravimetrically, while the purity was determined by HPLC.

## 3 RESULTS

### 3.1 Increasing precursor supply for improved CCM production

Previously, we constructed a CCM‐producing *S. cerevisiae* strain by expressing the 3‐DHS‐to‐CCM pathway and, after subjecting the strain to random mutagenesis, we selected the best performing mutant Mut131 by FACS via CCM‐responsive biosensor. We then increased the mutant strain's CCM‐producing ability by overexpressing the CCM biosynthesis genes and obtained ST8920 (Wang et al., 2020). The strain contains two copies of *PaAroZ* and *CaCatA* genes, three copies of *KpAroY* gene, 15 missense mutations, each leading to a non‐synonymous amino acid change, and one nonsense mutation, resulting in a stop‐codon and premature termination of an open reading frame (Table S1). We started with testing metabolic engineering strategies in this strain to improve the supply of precursor molecules, DAHP and PEP/E4P.

**Figure 2:**
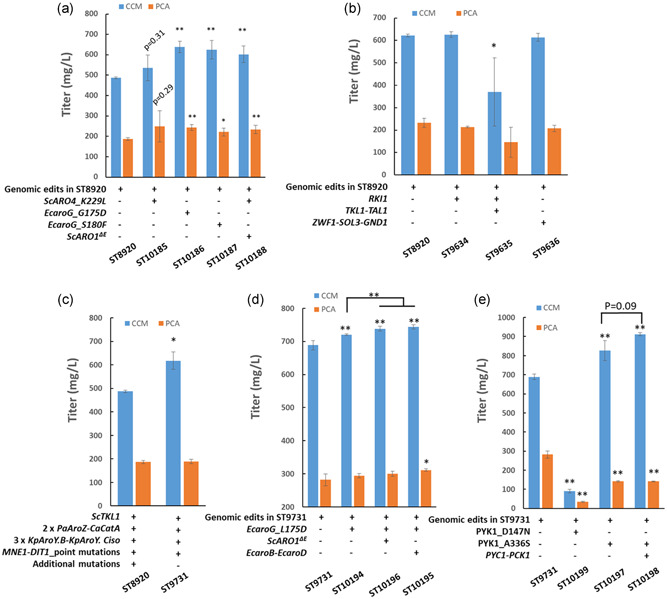
Improved CCM production by increasing the precursor supply. The engineered strains, derived from ST8920 (a, b) or ST9731 (c, d, and e), were cultivated in the mineral medium for 72 h. 50 mg/L uracil was supplemented when required. CCM concentration was quantified in the supernatant of the diluted cell culture. Data shown are mean values ± standard deviations (SDs) of triplicate. Statistical difference between control (ST8920 or ST9731) and indicated strains, as well as that between the indicated strains was determined by two‐tailed Student's *t*‐test (*p < 0.05, **p < 0.01)

To improve the supply of DAHP, we chromosomally expressed feedback‐resistant DAHP synthases from *S. cerevisiae* (*ScARO4* ^*K229L*^; Hartmann et al., 2003) and *E. coli* (*EcaroG* ^*L175D*^; Hu et al., 2003 or *EcaroG* ^*S180F*^; Ger et al., 1994) under strong constitutive promoters *PGK1p* and *TEF1p*, respectively. While *ScARO4* ^*K229L*^ expression (ST10185) did not significantly improve the CCM production, expression of *E. coli* DAHP synthases increased the CCM titer by 31% (ST10186 expressing *EcaroG* ^*L175D*^, 638 mg/L; Figure 2a) and 28% (ST10187 expressing *EcaroG* ^*S180F*^, 624 mg/L; Figure 2a).

We then sought to increase E4P supply by constitutively overexpressing genes from the non‐oxidative (*RKI1*, *TKL1*, and *TAL1*) or oxidative (*ZWF1*, *SOL3*, and *GND1*) pentose phosphate pathway (PPP) branches. Introducing *RKI1* or *ZWF1*‐*SOL3‐GND1* under strong constitutive promoters into ST8920 did not change the CCM titer, whereas the overexpression of *RKI1*‐*TKL1*‐*TAL1*, unexpectedly, reduced the CCM production by 40% (Figure 2b). We, therefore, did not continue with the PPP flux manipulation.

As strain ST8920 was derived from a mutagenized strain and contained 15 missense mutations and 1 nonsense mutation, we were concerned if some of the mutations would have impaired the strain's growth, CCM production, or other physiological parameters. In our previous reverse engineering work, we reconstructed a strain RC3, which bears two copies of *PaAroZ* and *CaCatA*, one copy of *KpAroY*, and two missense mutations beneficial for CCM production (Wang et al., 2020). To obtain the same copy number of the CCM pathway as in strain ST8920, we added two more copies of *KpAroY* that encodes PCA decarboxylase into RC3, obtaining strain ST9731. The resulting ST9731 strain produced 27% more CCM than ST8920 (Figure 2c). We, therefore, continued with engineering ST9731 strain.

We chromosomally integrated *EcaroG* ^*L175D*^ under the TEF1p into ST9731, and the resulting strain ST10194 produced 4.7% more CCM, 721 mg/L (Figure 2d). We then sought to pull more DAHP to 3‐DHS by overexpressing 3‐DHQ synthase and 3‐dehydroquinase in ST10194. In *S. cerevisiae*, a penta‐functional AROM protein, Aro1p, drives the multistep conversion of DAHP to 5‐enolpyruvylshikimate‐3‐phosphate (EPSP) via 3‐DHS (Figure 1). The E‐subunit (Aro1p_E) encodes shikimate dehydrogenase that catalyzes 3‐DHS conversion into shikimate and, thus, diverts 3‐DHS into the aromatic amino acid biosynthesis (Figure 1). To increase the conversion of DHAP into 3‐DHS, we expressed AROM protein variant lacking the shikimate dehydrogenase domain (*ScARO1* ^ΔE^). In parallel, we also tested the expression of 3‐DHQ synthase EcaroB and 3‐dehydroquinase EcaroD from *E. coli*. These two enzymes together also catalyze the conversion of DAHP into 3‐DHS. These modifications increased CCM titer to 738 mg/L (in ST10196) and 744 mg/L (in ST10195; Figure 2d).

We also attempted to increase PEP supply by de‐activating the pyruvate kinase (PYK) by introducing two point‐mutations (D147N and A336S) into Pyk1p (Hassing et al., 2019), which converts PEP to pyruvate. These strategies were tested on strain ST9731. D147N mutation decreased the biomass formation by 91% (Figure S1) and CCM titer by 87% (ST10199, Figure 2e), but A336S mutation enhanced the CCM titer by 20% (Figure 2e, 826 mg/L by ST10197) without affecting the final OD_600_ (Figure S1). We also tested if it is possible to re‐divert pyruvate to supply more PEP by constitutively expressing pyruvate carboxylase (*PYC1*) and PEP carboxykinase (*PCK1*). Indeed, the resulting strain, ST10198, expressing *PYC1* and *PCK1* under strong promoters *TEF1p* and *PGK1p*, produced 912 mg/L CCM (Figure 2e). These results suggested that enhancing PEP supply was beneficial for the improvement of CCM production in *S. cerevisiae* CEN.PK strains.

### 3.2 Engineering shikimate dehydrogenase for improved CCM production

**Figure 3:**
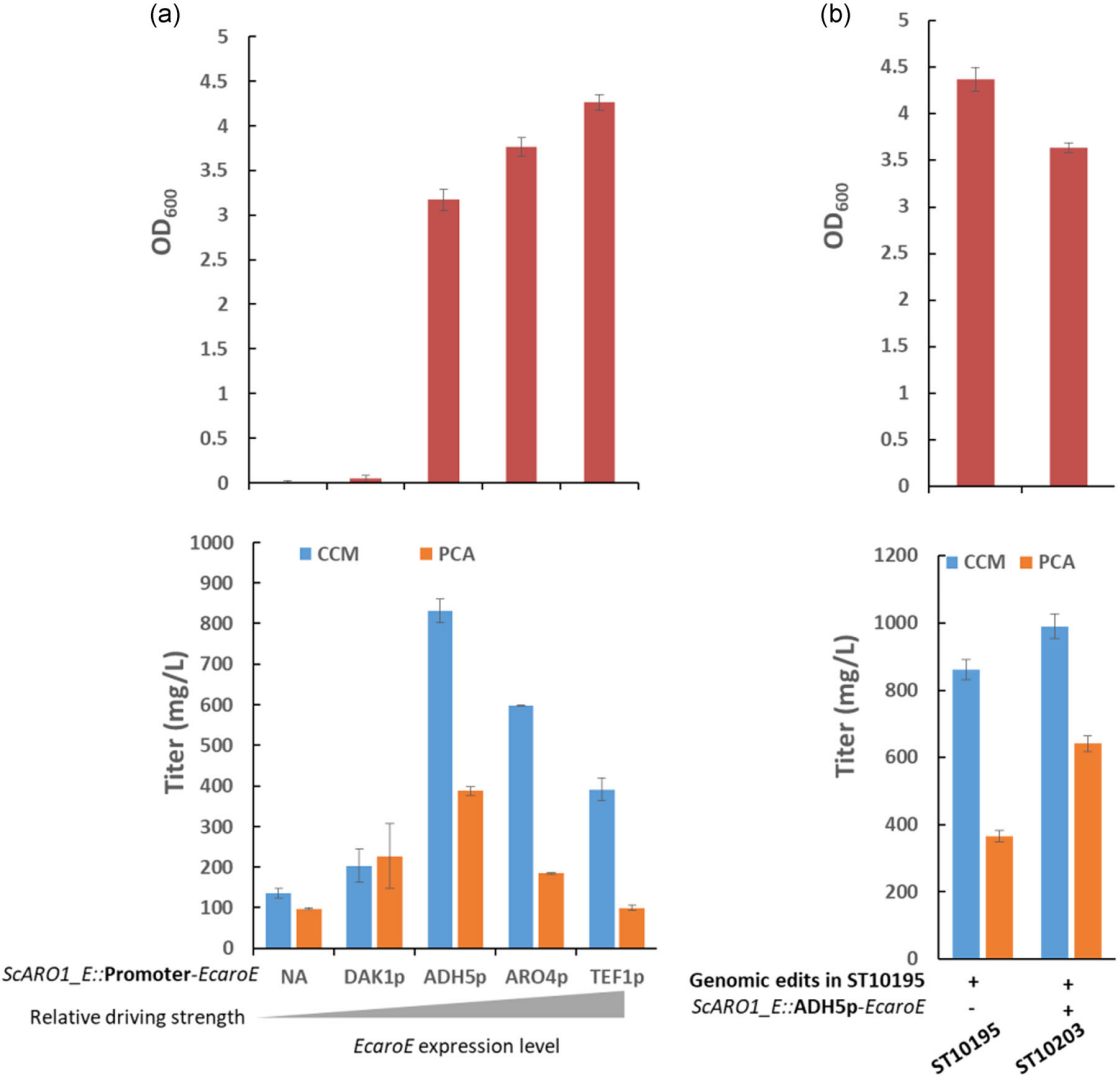
Improved CCM production by fine‐tuning of shikimate dehydrogenase. The engineered strains that carry *EcaroE* under different promoters in *ScARO1_E* deleted ST10195 (a, b) were cultivated in the mineral medium for 72 h. 50 mg/L uracil was supplemented when required. OD_600_ of the cell culture was quantified. CCM concentration was quantified in the supernatant of the diluted cell culture. Data shown are mean values ± SDs of triplicate.

In the engineered strains, 3‐DHS is either converted to CCM via a 3‐step reaction or to shikimate by Aro1p_E. We, therefore, aimed to fine‐tune the metabolic flux to shikimate and enforce more 3‐DHS available for CCM synthesis, using ST10195, a strain with a strengthened DHAP‐to‐3‐DHS flux, as the parental strain. We replaced the *ARO1_E* (4036–4767 bp of the *ARO1*) with a stop codon and CYC1t, and in the meantime, introduced *aroE* from *E. coli* (*EcaroE*), which encodes a shikimate dehydrogenase spatially separate from the native Aro1p^ΔE^. *EcaroE* gene was expressed under the control of four different promoters with ascending driving force, DAK1p (0.5%), ADH5p (3.1%), ARO4p (14%), and TEF1p (100%, relative ratio was calculated based on the GFP expression levels driven by the respective promoters in synthetic medium; Keren et al., 2013). The *ARO1_E* deletion strain cannot synthesize aromatic amino acids and thus could not grow in a mineral medium (Figure 3a). The abolished cell growth was rescued by expression of *EcaroE*, to an extent corresponding to the driving forces of the promoters: a final OD_600_ of 0.05, 3.2, 3.8, and 4.3 were obtained, respectively (Figure 3a). In contrast to the consistently restored biomass, the highest CCM titer of 831 mg/L was achieved in ST10203 (ADH5p), in comparison to 203 mg/L in ST10202 (DAK1p), 598 mg/L in ST10204 (ARO4p), and 391 mg/L in ST10205 (TEF1p) (Figure 3a). ST10203 showed a 15% and 76% higher CCM and PCA titer than the control strain ST10195, which expresses the intact native *ARO1* (Figure 3b). These results indicated that a modulated weak expression of a spatially dispersed shikimate dehydrogenase could improve the CCM production in *S. cerevisiae*.

### 3.3 Combination of the beneficial metabolic engineering strategies further enhance the CCM production

**Figure 4:**
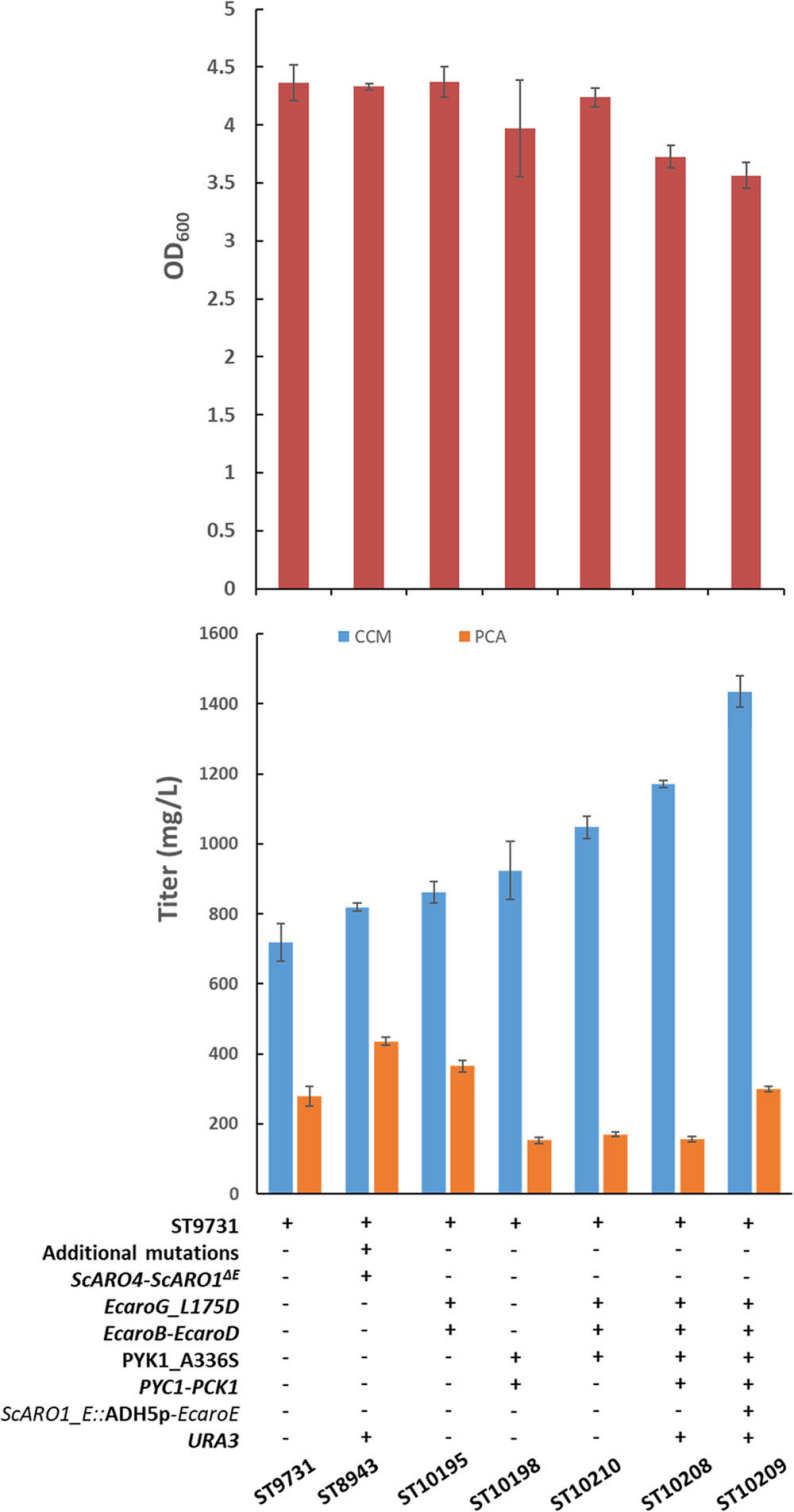
CCM production of engineered strains on mineral medium. The engineered strains derived from ST9731 were cultivated in the mineral medium for 72 h. ST8943, the strain that showed the highest titer in our previous report, was also tested for comparison. 50 mg/L uracil was supplemented when required. CCM concentration was quantified in the supernatant of the diluted cell culture. Data shown are mean values ± SDs of triplicate.

Aiming for an engineered strain towards improved CCM production, we iteratively combined the beneficial genetic manipulations identified above (Figure 4). Combination of all beneficial genetic manipulations—constitutively strong expression of *EcaroG* ^*L175D*^, *EcaroB, EcaroD*, *PYC1*, and *PCK1*, *PYK1* ^*A336S*^, and modulated *EcaroE* expression (*ARO1_E*::ADH5p‐*EcaroE*)—generated the most productive strain, ST10209. By cultivation in the mineral medium for 72 h, the strain accumulated less biomass (OD_600_ is 3.6 vs. 4.4 of ST9731) but produced 1.4 g/L CCM in comparison to 819 mg/L by ST8943 (Figure 4), a strain that produced 20.8 g/L CCM through controlled fed‐batch fermentation as the highest titer in our previous report (Wang et al., 2020).

When the two strains were cultivated on FIT medium, a simulated fed‐batch medium with a higher glucose supply (60 vs. 20 g/L in mineral medium) but without pH control either (Wang et al., 2020), ST10209 produced CCM at the same level as ST8943, 2.2 g/L CCM (Figure S2). It is possible that, when pH was uncontrolled, CCM is highly toxic (Wang et al., 2020) and, therefore, may have inhibited the cellular activity towards a higher CCM production. Nevertheless, the final biomass concentration of ST10209 was 26% lower than ST8943, and the specific CCM yield (mg/OD_600_) was 35% higher (Figure S2). The strain ST10209 may have re‐directed some carbon from biomass formation to CCM synthesis. The full potential of this strain needs exploring via controlled fed‐batch fermentation.

### 3.4 Controlled fed‐batch fermentation and scale‐up

**Figure 5:**
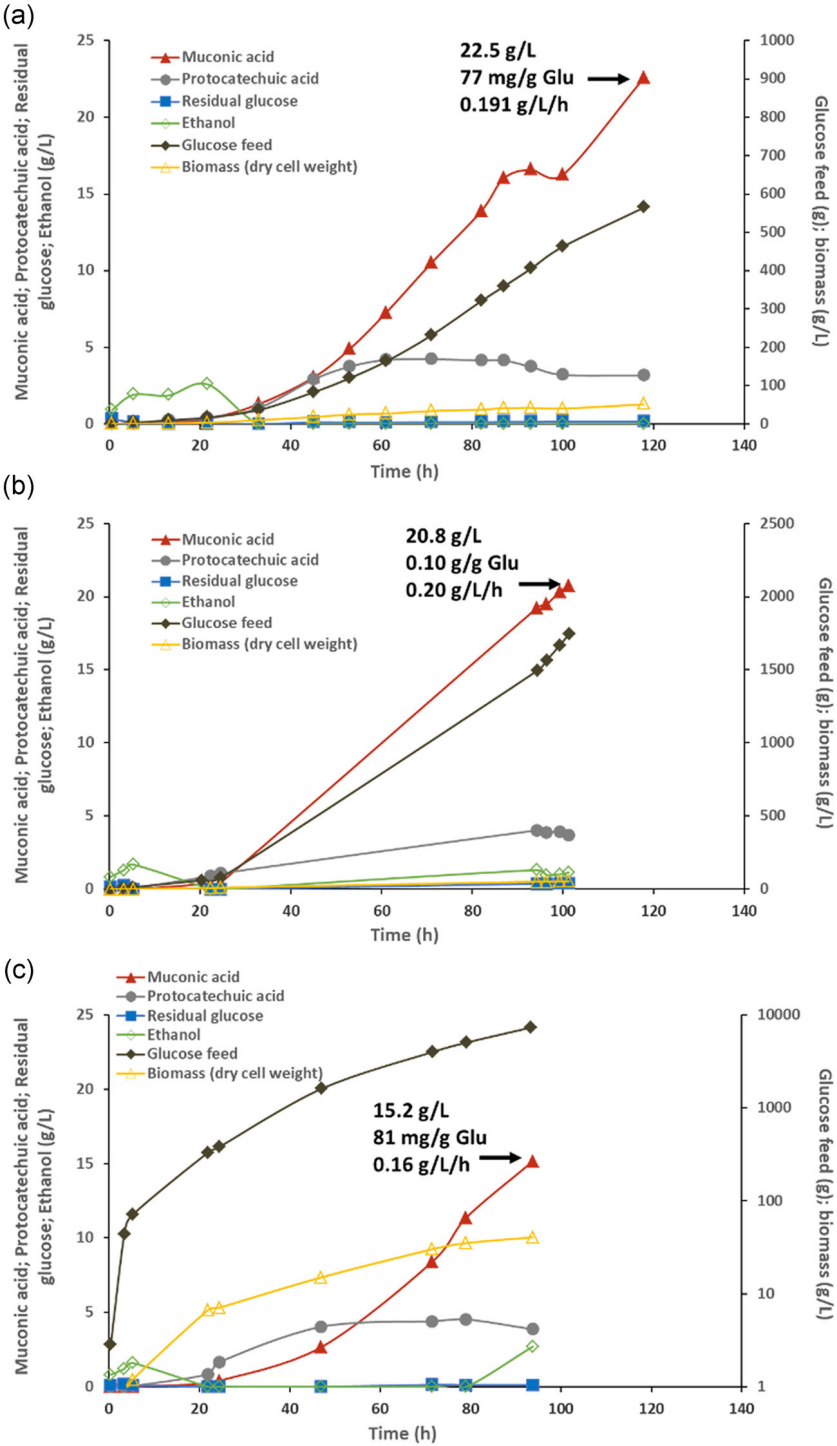
Controlled fed‐batch fermentation for CCM production. Fermentations were performed in 2 L (a), 10 L (b), and 50 L (c) fermenters with a starting fermentation broth of 1.3, 5, and 25 L, respectively. CCM titers (g/L), yields (mg/g glucose (Glu)) and productivities (mg/L/h) indicated were for 117.8, 101.25, and 93.75 h, respectively. They represented the highest production metrics achieved in the individual run. The fermentation of both 2 and 10 L fermenters was ended when the agitation reached the highest rate, while that of the 50 L fermenter was ended earlier due to its high demand. The 10 L fermenter (b) fermentation was performed over the weekend/holiday, and manual sampling was not carried out between 25 and 94 h. Data shown are from a single replicate.

In previous work, we have obtained the highest CCM production metrics for yeast: a titer of 20.8 g/L, an overall yield of 66.2 mg/g glucose, and a volumetric productivity of 0.14 g/L/h, after 149 h of fermentation of ST8943 strain in 2 L fermenter (Wang et al., 2020). Using the same fed‐batch strategy to keep the glucose concentration limiting, we cultivated the ST10209 in a 2 L fermenter and achieved a slightly higher CCM titer (22.5 g/L) at 117.80 h with a much lower biomass concentration (51 g/L dry cell weight for ST10209 vs. 69 g/L for ST8943; Figure 5a and Table 1). The CCM yield (76.7 mg/g glucose) and productivity (191.3 mg/L/h; Table 1), in comparison to those of ST8943, were improved by 16% and 38%, respectively. The improvement of CCM titer was not that big. This can be possibly explained by limitations of oxygen transfer (Figure S3) and the abiotic stress caused by high CCM concentration at the late fermentation stages.

**Table 1:** Production metrics of strain ST10209 fermented at 2, 10, or 50 L scale

Scales	Time points (h)	Production metrics		
		Titer (g/L)	Yield (mg/g glucose)	Productivity (mg/L/h)
2 L	92.97	16.6	67.1	178.5
	117.80	22.5	76.7	191.3
10 L	94.25	19.2	101.7	204.0
	101.25	20.8	99.9	204.9
50 L	93.75	15.2	81.3	162.4

To test the scalability of the CCM production bioprocess using the engineered strain ST10209, we cultivated the ST10209 strain in both 10 and 50 L fermenters. In a 10 L fermenter, a final dry cell weight of 53.2 g/L and a final CCM titer of 20.8 g/L were reached at 101.25 h, resulting in a CCM yield of 0.1 g/g glucose and an overall volumetric CCM productivity of 0.205 g/L/h (Figure 5b and Table 1). We ran a shorter cultivation in a 50 L fermenter, and achieved a dry cell weight of 40.5 g/L and a CCM titer of 15.2 g/L at 93.75 h (Figure 5c and Table 1). The resulting CCM yield and productivity were 81.3 mg/g glucose and 162.4 mg/L/h (Figure 5c and Table 1), respectively. In both tests, the titers, yields, and productivities are higher or comparable to those obtained in the 2 L fermenter, indicating the established bioprocess is robust for scale‐up.

### 3.5 CCM recovery and purification

**Figure 6:**
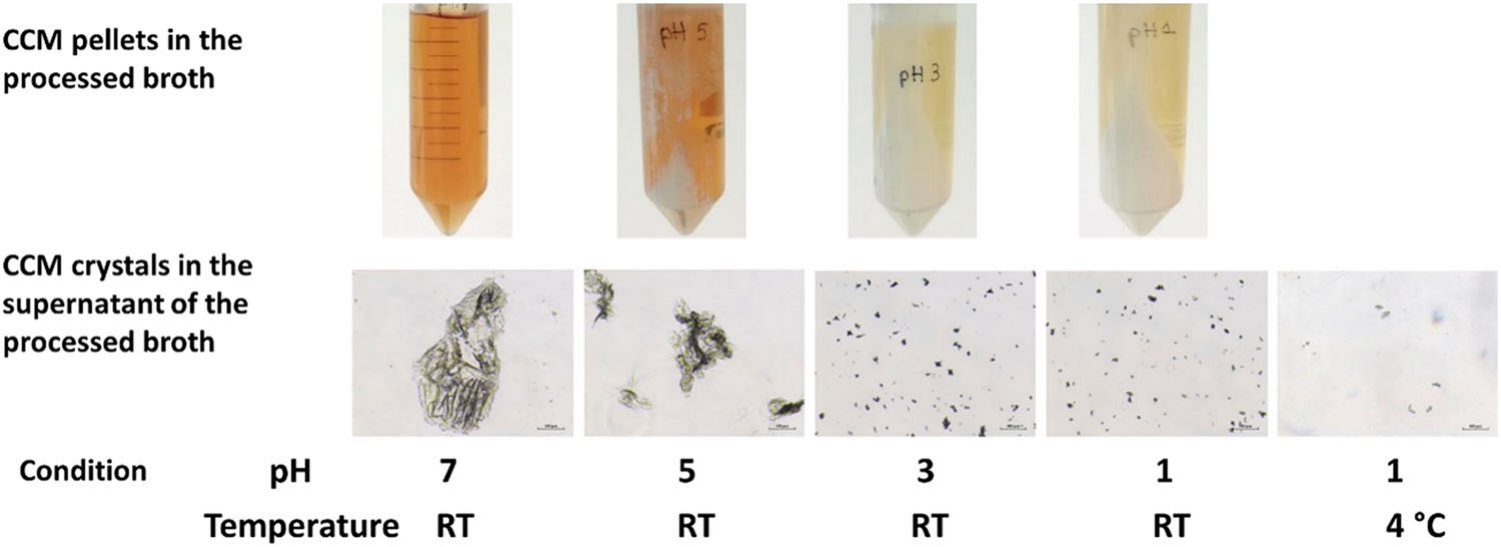
Precipitation of muconic acid and microscopic observation of the crystals in the supernatant. The pH of fermentation broth was modified to 7, 5, 3, or 1 at room temperature, or to 1 after cooling at 4°C. The bar on the micrographs corresponds to 100 µm

We sought to develop a process to recover and purify CCM from the broth of yeast fermentation. It is known that CCM is in its protonated form at pH 3.64 and shows a low solubility. Therefore, a low pH could enable the CCM precipitation and facilitate CCM recovery. The exposure of CCM to pH lower than 6 could render the irreversible conversion of CCM into *cis*, *trans* form (Carraher et al., 2017). Such conversion could be inhibited under a low temperature. The calculated isomerization rate is 7.6 × 10^−7^ s^−1^ at 4°C, almost an order of magnitude lower than at room temperature. We first tested the effect of pH and temperature on the precipitation of muconic acid. At pH 7 and room temperature, the fermentation broth was in general clear by eye and no precipitation was visible, while large CCM crystals (ca. 500 µm in length) can be observed in the broth via microscopy (Figure 6). When the broth was acidified through the addition of sulfuric acid, the formation of a white precipitate was observed, and crystalline materials turned to a larger number of smaller particles (generally < 50 µm) as pH drops to 1 via 5 and 3 (Figure 6). When the acidified broth is cooled to 4°C, the supernatant becomes mostly clear under the microscope, indicating that the precipitation was enhanced by the low‐temperature treatment. We then determined to precipitate the CCM by adjusting the pH below 2 and incubating the broth at 4°C.

We then used 6 L fermentation broth from the 10 L fermentation to evaluate the process of CCM recovery and purification. The total CCM amount was 123.3 g. We employed a treatment with activated carbon to remove the impurity and recover the CCM by precipitation at 4°C and pH below 2. 61.7 g of CCM were recovered, equivalent to a recovery yield of 50%. The CCM purity was 96.0%. To increase the recovery yield, we re‐suspended the CCM adsorbed on the activated carbon in reverse osmosis water and precipitated the CCM. The washing and precipitation were carried out twice, recovering 12.7 g of CCM with a purity of 94.2% in the first round and 7.29 g of CCM with 92.7% purity in the second round. The overall CCM recovery yield was 66.3%.

The progressive decrease in purity reflects the fact that other compounds which had been adsorbed were also released in each washing step of the activated carbon. When pooling all fractions of recovered CCM, an overall purity of 95.4% was obtained with low content of the *cis*, *trans* isomer (<5%). The presence of *cis, trans*‐isomer is the result of isomerization (isomerization rate peaked at pH ca. 4 and high temperature) of biologically produced *cis, cis*‐isomer during the fermentation, purification, and recovery processes.

## 4 DISCUSSION

In this study, we developed a yeast‐based process for CCM production by applying systematic strain engineering, controlled fed‐batch fermentation and scale‐up to 50 L, and CCM recovery and purification.

By enhancing PEP supply and rewiring the shikimate pathway (both increasing the total metabolic flux and reallocating the flux at the 3‐DHS node), we doubled the CCM titer (ST10209 vs. ST9731, Figure 4). Pyk1p converts PEP to pyruvate, while Pyc1p and Pck1p catalyze the conversion of pyruvate to PEP through oxaloacetic acid. Pyk1p de‐activation by A336S mutation and overexpression of *PYC1* and *PCK1* improved CCM titer by 33% (Figure 2e). The beneficial impact of *PYK1* mutation is consistent with the previous study where A336S and D147N mutations improve the production of 2‐phenylethanol (Hassing et al., 2019), an aromatic amino acid‐derived compound in *S. cerevisiae*. These results together indicated that limiting PEP‐to‐pyruvate conversion and redirecting pyruvate to PEP could offer more PEP for the synthesis of the shikimate pathway (intermediates)‐derived products in CEN.PK background strains. Constitutively strong expression of *E. coli* genes for DAHP synthase (*EcaroG* ^*L175D*^), 3‐dehydroquinate synthase (*EcaroB*), and 3‐dehydroquinase (*EcaroD*) increased the CCM titer by 8.1% (Figure 2d). The three enzymes catalyze the condensation of PEP and E4P to 3‐DHS via DAHP and 3‐DHQ. These results confirm that forcing carbon flux to shikimate pathway is a robust strategy to improve the production of shikimate pathway derivative (Leavitt et al., 2017; Liu et al., 2019; Rodriguez et al., 2015). Fine‐tuning the *E. coli* shikimate dehydrogenase (*EcaroE*) expression in the *ARO1_EΔ* background strains could substantially re‐distribute the carbon flux around 3‐DHS. The essential shikimate dehydrogenase would then become spatially separate from the four enzymes of Aro1p_ΔE for shikimate pathway. It could potentially thwart the flux from 3‐DHS to shikimate. Moreover, the moderately low *EcaroE* expression further reduced the flux to shikimate, and, therefore, limited biomass accumulation (Figure 3). In the meantime, this could provide more 3‐DHS for CCM biosynthesis and enable a 15% and 76% improvement of CCM and PCA titer (Figure 3b). This strategy may be generally applicable to improve the production of 3‐DHS derived products.

Previous studies and our results suggest that the effect of genetic manipulations depends on the strain background and the genetic context. Overexpression of *PYC1* and *PCK1* improved the CCM production in our CEN.PK 113‐7D‐derived strain (Figure 2e); while it significantly decreased the CCM production by more than 60% in InvSc1 (diploid)‐derived strains (Suastegui, Matthiesen, et al., 2016). *RKI1* overexpression resulted in 47% titer improvement of muconic acid and PCA in BY4741‐derived strain (Suástegui et al., 2017), but had no impact in our strain (Figure 2b). The discrepancy is likely because of the strain‐dependent regulation of flux distribution. It was noted that upon the same engineering towards shikimate accumulation, four *S. cerevisiae* strains, YSG50, BY4743, BY4741, and INVSc1, showed distinct flux distribution through glycolysis and PPP, and produced shikimate at very different levels: from 30 to 350 mg/L (Suastegui, Guo, et al., 2016). The metabolic architecture of the wild‐type strain may also be modified differently by intensive strain engineering. Pyk1p^D147N^ only moderately slowed down the growth rate (34%) while dramatically increased 2‐phenylethanol production (85%) in CEN.PK 113‐7D‐derived strain (Hassing et al., 2019). However, introducing the same mutation into a CEN.PK 113‐7D‐derived strain for CCM production nearly abolished both the growth (91% decrease in final biomass) and CCM production (87% decrease; Figures 2e and S1). In contrast to this, another mutation, A336S, which reduced Pyk1p activity less dramatically than D147N, enhanced both 2‐phenylethanol (53%; Hassing et al., 2019) and CCM production (20%, Figure 2e). Our data highlighted the significance of strain selection and context‐based metabolism rewiring in developing efficient cell factories.

Previously, we reported the highest titer, yield, and productivity of *S. cerevisiae* CCM cell factories. A maximum CCM concentration of 20.8 g/L and overall volumetric productivity of 0.14 g/L/h after 149 h of fermentation, with a maximum productivity of 0.15 g/L/h attained at 123.5 h of fermentation, at which time the CCM concentration was 19.0 g/L (Wang et al., 2020). Our results with the improved strain show an 8% increase in titer (22.5 g/L), but more importantly, a 16% increase in yield (76.7 mg/g glucose) and a 38% increase in productivity (0.19 g/L/h). The improvement in yield and productivity in the 10 L fermenter were even higher: 51% (0.1 g/g glucose) and 47% (0.20 g/L/h), respectively. By enabling the production in less fermentation time and volume per cycle, both investment and operational costs are significantly reduced. Additionally, in our previous work, the cell concentration was 70.4 g/L for a CCM titer of 20.8 g/L, leading to a specific production of 0.296 g CCM/g dry cell weight, while the current results highlight a significantly higher specific production 0.438 g CCM/g dry cell weight (48% increase). The bioprocess developed using the improved strain was robust in scale‐up (Figure 6). It is noteworthy that all improvements were obtained using the exact same fermentation strategy as in our previous work, highlighting not only the impact of continued strain improvement in the increase of the competitiveness of existing processes, but also validating the efficiency of an approach addressing simultaneously strain and process improvement instead of using a sequential approach of first improving the strain and only after addressing the process.

In this study, the successful recovery of CCM was achieved by simple activated carbon adsorption and precipitation processes with purities above 95%, with a content of *cis*, *trans* isomer lower than 5%. The purification yield was 66.3%, while the main CCM losses were the filtrates obtained after the CCM precipitation (17 g out of 123.3 g in this study). In a large‐scale production facility, such fractions could, at least partly, be recirculated, potentially increasing the yield up to 80.9% and the activated carbon could be reused in successive purification cycles to minimize losses of CCM, contributing to further increasing the overall yield of the process (Figueira et al., 2017).

In summary, we reprogrammed the CCM‐producing *S. cerevisiae* strain by increasing PEP supply and rewiring the shikimate pathway. Based on this strain, we established a CCM bioproduction process that is robust in the scale‐up, achieving the to‐date highest CCM production metrics in yeasts: a titer of 22.5 g/L (2 L fermenter), a yield of 0.1 g/g glucose, and a productivity of 0.2 g/L/h (10 L fermenter). We developed the first recovery and purification process for yeasts, enabling a CCM recovery yield of 66.3% with 96.3% purity.

## CONFLICT OF INTERESTS

The authors declare that there are no conflict of interests.

## AUTHOR CONTRIBUTIONS

Guokun Wang, Irina Borodina, and Bruno S. Ferreira conceived and designed the study. Guokun Wang, Aline Tavares, Simone Schmitz, Lucas França, Hugo Almeida, João Cavalheiro, Ana Carolas, and Süleyman Øzmerih performed the experiments. Guokun Wang, Simone Schmitz, Bruno S. Ferreira, and Irina Borodina analyzed the data. Lars M. Blank, Bruno S. Ferreira, and Irina Borodina supervised the project. Guokun Wang, Bruno S. Ferreira, and Irina Borodina wrote the manuscript, and all authors read, edited, and approved the final manuscript.

## Supporting information

Supporting information.

## Data Availability

The data that support the findings of this study are available from the corresponding author upon reasonable request.
