# Supplementary material for: An integrated yeast‐based process for cis,cis‐muconic acid production
Source: Biotechnol Bioeng. 2021 Nov 24;119(2):376–87. doi: 10.1002/bit.27992 (PMC9299173; doi:10.1002/bit.27992)
Supplement: Supplementary file 1 — Supporting information. [file BIT-119-376-s001.docx]

**Supplementary information**

An integrated yeast-based process for *cis*,*cis*-muconic acid production

Guokun Wang^1,4^, Aline Tavares^2^, Simone Schmitz^1,3^, Lucas França^2^, Hugo Almeida^2^, João Cavalheiro^2^, Ana Carolas^2^, Süleyman Øzmerih^1^, Lars Blank^3^, Bruno S. Ferreira^2,^ ^*^, Irina Borodina^1,*^

1 The Novo Nordisk Foundation Center for Biosustainability, Technical University of Denmark, DK-

2800 Kgs. Lyngby, Denmark

2 Biotrend - Inovação e Engenharia em Biotecnologia SA, 3060-197 Cantanhede, Portugal

3 Institute of Applied Microbiology-iAMB, Aachen Biology and Biotechnology-ABBt, RWTH Aachen University, Worringer Weg 1, Aachen, 52074, Germany

4 Tianjin Institute of Industrial Biotechnology, Chinese Academy of Sciences, Tianjin, 300308, China

*Correspondence:

Professor Irina Borodina,

The Novo Nordisk Foundation Center for Biosustainability, Technical University of Denmark,

Kemitorvet Building 220, 2800 Kongens Lyngby, Denmark.

irbo@biosustain.dtu.dk

Dr. Bruno S. Ferreira

Biotrend - Inovação e Engenharia em Biotecnologia SA, 3060-197 Cantanhede, Portugal

bsferreira@biotrend.pt


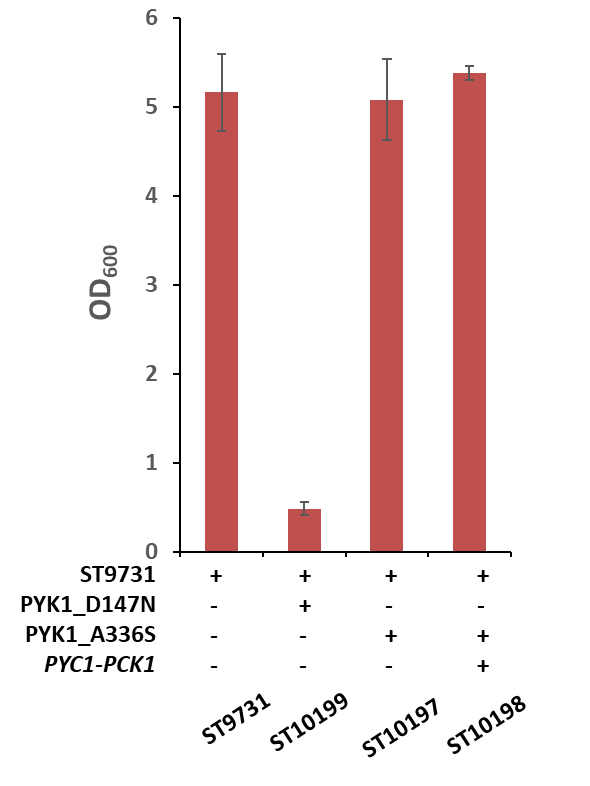


Fig. S1 OD600 of 72 h culture of engineered strains

Cells were cultivated on mineral medium supplemented with 50 mg/L uracil. Data shown are mean values ± SDs of triplicate.


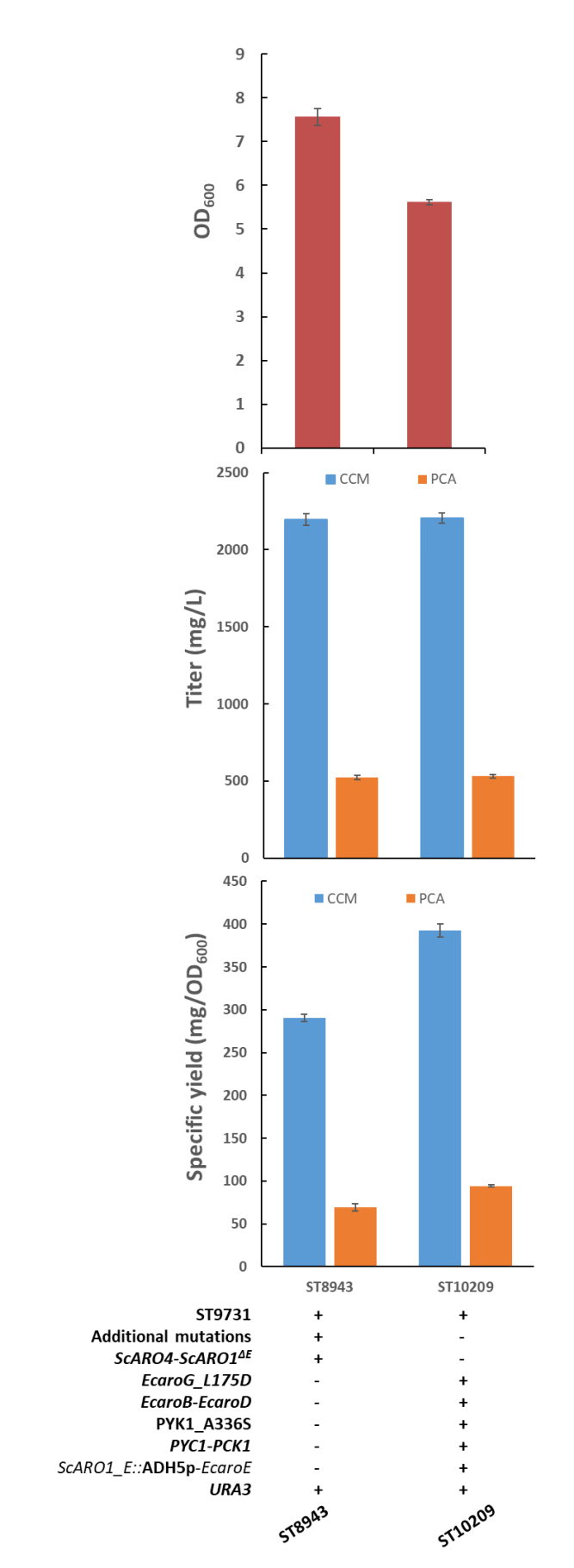


Fig. S2 Biomass accumulation and CCM production in strains ST8943 and ST10209 in mimicked fed-batch medium

Cells were cultivated in fed-in-time medium. OD600 and metabolite were quantified after cultivation for 72 h. Data shown are mean values ± SDs of triplicate.


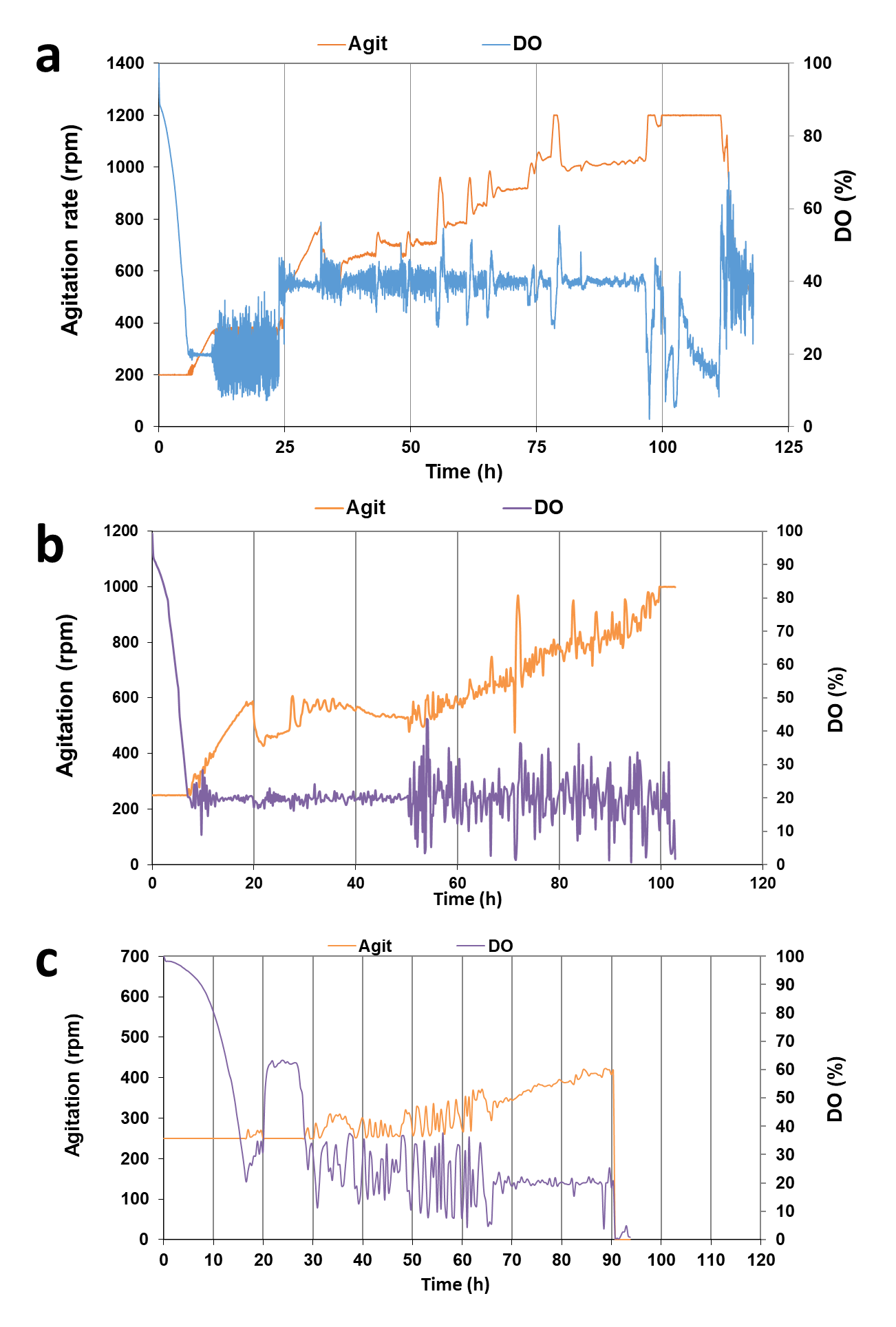


Fig. S3 Fermentation parameters during the controlled fed-batch fermentations in 2 L (a), 10 L (b), and 50 L (c) fermenters

Table S1 Strains used in this work

| Strain | Genotype | Reference |
| --- | --- | --- |
| CEN.PK113-7D | MAT a URA3 HIS3 LEU2 TRP1 | [^1^](#_ENREF_1) |
| CEN.PK102-5B | MATa ura3-52 his3∆1 leu2-3/112 MAL2-8c SUC2 | [^1^](#_ENREF_1) |
| ST8920 | CEN.PK102-5B X-4-HIS5-ScTKL1-KpAroY.D XI-1-KlLEU2-PaAroZ-CaCatA XII-2-amdSYM-Cas9 X-2-KpAroY.B-KpAroY.Ciso X-3-REV1p-BenM variant MP02_D04-XI-2-CYC1p-yEGFP XI-5-AroY.B-AroY.Ciso-XII-4-AroY.B-AroY.Ciso PWP2_Ser388Phe EST2_Val182Met RET2_Pro15Ser IMA3_Asp242Asn ATG1_Glu382Lys PET54_Gly19Glu OCA5_Ala649Val DIT1_Glu526Gly SAP1_Pro20Leu PUT3_Ser615Phe NUD1_Arg819Trp KNS1_Ser227Leu CDC15_Pro429Phe CTS2_Gly497Glu MNE1_Lys450Glu NUP53_Ser69* PaAroZ-CaCatA-KlLEU2 | [^2^](#_ENREF_2) |
| ST8943 | ST8920-XI-3-ScAro4-ScAro1deltaAroE-KlURA3 | [^2^](#_ENREF_2) |
| ST9659/RC3 | CEN.PK102-5B X-4-HIS5-ScTKL1-KpAroY.D-XI-1-KlLEU2-AroZ-CaCatA-amdSYM-Cas9-X-2-AroY.B-AroY.Ciso-X-3-REV1p-BenM variant MP02_D04-XI-2-CYC1p-yEGFP MNE1_ Lys450Glu DIT1_ Glu526Gly XII-5-PaAroZ-CaCatA | [^2^](#_ENREF_2) |
| ST9634 | ST8920-XII-5-RKI1 | This study |
| ST9635 | ST8920-XII-5-RKI1-TKL1-TAL1 | This study |
| ST9636 | ST8920-XII-1-ZWF1-SOL3-GND1 | This study |
| ST9731 | ST9659-XI-5-AroY.B-AroY.Ciso and XII-4-AroY.B-AroY.Ciso | This study |
| ST10185 | ST8920-XI-2-ScARO4_K229L | This study |
| ST10186 | ST8920-XI-2-EcaroG_L175D | This study |
| ST10187 | ST8920-XI-2-EcAroG_S180F | This study |
| ST10188 | ST8920-XI-2-ScARO1_ΔE-ScARO4_K229L | This study |
| ST10194 | ST9731-XI-2-EcaroG_L175D | This study |
| ST10195 | ST9731-XI-2-EcaroG_L175D-XI-3-EcaroB-EcaroD | This study |
| ST10196 | ST9731-XI-2-EcaroG_L175D-XI-3-EcaroB-EcaroD-XI-3-ScARO1_ΔE | This study |
| ST10197 | ST9731-PYK1_A336S | This study |
| ST10198 | ST9731-PYK1_A336S-PYC1-PCK1 | This study |
| ST10199 | ST9731-PYK1_D147N | This study |
| ST10202 | ST10195-ScARO1_E::DAK1p-EcaroE | This study |
| ST10203 | ST10195-ScARO1_E::ADH5p-EcaroE | This study |
| ST10204 | ST10195-ScARO1_E::ARO4p-EcaroE | This study |
| ST10205 | ST10195-ScARO1_E::TEF1p-EcaroE | This study |
| ST10206 | ST10195-ScARO1_EΔ | This study |
| ST10207 | ST10198-XI-2-EcaroG_L175D-XI-3-EcaroB-EcaroD | This study |
| ST10208 | ST10207-URA3::KlURA3 | This study |
| ST10209 | ST10207-URA3::KlURA3 ScARO1_E::ADH5p-EcaroE | This study |
| ST10210 | ST10195-PYK1_A336S | This study |

Table S2 Plasmids used in this work

| Plasmid | Parental plasmid | Added module | Constitution | Note | Construction approaches/reference |
| --- | --- | --- | --- | --- | --- |
| pQC003/pCFB8904 | NA | NA | 2µ ori, KlURA3, AmpR, GAL80 gRNA | pMEL10 derived plasmid, used as template for backbone preparation for the plasmid via PCR, for the mutation introduction | [^3^](#_ENREF_3) |
| pCFB3053 | NA | NA | 2µ ori, AmpR, NatMXsyn gRNA X-2 XI-5 XII-4 | gRNA plasmid for simultaneous integration into X-2, XI-5 and XII-4 sites | [^4^](#_ENREF_4) |
| pCFB3050 | NA | NA | 2μ ori, AmpR,NatMXsyn, XII-5-gRNA | gRNA plasmid for XII-5 integration | [^4^](#_ENREF_4) |
| pCFB3047 | NA | NA | 2μ ori, AmpR,NatMXsyn, XII-1-gRNA | gRNA plasmid for XII-1 integration | [^4^](#_ENREF_4) |
| pCFB9940 | pQC003 | yEGFP-gRNA | 2µ, ori, KlURA3, AmpR, yEGFP-gRNA | Introducing double strand break at yEGFP locus | Gibson assembly |
| pCFB9941 | pQC003 | ScARO1_E -gRNA | 2µ, ori, KlURA3, AmpR, ScARO1_E -gRNA | Introducing double strand break at ScARO1_E locus | Gibson assembly |
| pCFB9942 | pQC003 | gRNA-donor for PYK1_A336S | 2µ, ori, KlURA3, AmpR, gRNA-donor for PYK1_A336S | Introducing missense mutation in PYK1 | Gibson assembly |
| pCFB9943 | pQC003 | gRNA-donor for PYK1_D147N | 2µ, ori, KlURA3, AmpR, gRNA-donor for PYK1_D147N | Introducing missense mutation in PYK1 | Gibson assembly |
| pCFB9944 | pQC003 | BenM-gRNA | 2µ, ori, KlURA3, AmpR, BenM-gRNA | Introducing double strand break at BenM locus | Gibson assembly |
| pCFB8337 | NA | NA | ori, AmpR, X-3-MsEgtD<-pPGK1-pTEF1->MsEgtB | Plasmid for marker free integration into X-3 site, used as template for backbone and double promoter preparation | [^5^](#_ENREF_5) |
| pCFB9945 | pCFB8337 | ScPYC1-TEF1p-PGK1p-ScPCK1 | ori, AmpR, X-3-CYC1t-ScPYC1-TEF1p-PGK1p-ScPCK1-ADH1t | Overexpression of PYC1 and PCK1 | USER cloning |
| pCFB2696 | NA | NA | ori, AmpR, XI-3-KlURA3-KpAroY.B-KpAroY.Ciso | Template for integrative cassettes (XI-5-AroY.B-Ciso, XII-4-AroY.B-Ciso) construction | [^6^](#_ENREF_6) |
| pCFB1075 | NA | NA | ori, AmpR, X-4-LoxP-SpHiS5 EcaroG_L175D | Template for PCR amplification of EcaroG_L175D expression cassette | [^7^](#_ENREF_7) |
| pCFB1076 | NA | NA | ori, AmpR, X-4-LoxP-SpHiS5 EcAroG_S180F | Template for PCR amplification of EcAroG_S180F expression cassette | [^7^](#_ENREF_7) |
| pCFB1955 | NA | NA | ori, AmpR, XI-1-LoxP-KlLEU2 EcaroB - EcaroD | Template for PCR amplification of EcaroB - EcaroD expression cassette | [^7^](#_ENREF_7) |
| pCFB9114 | NA | NA | ori, AmpR, X-4-ScAro4pm_K229L-ScAro7pm | Template for PCR amplification of ScAro4pm_K229L expression cassette | [^8^](#_ENREF_8) |
| pCFB8808 | NA | NA | ori, AMP, XI-3-TEF1p-ScARO4-PGK1p-ScARO1ΔE-KlURA3 | Template for PCR amplification of ScARO1_ΔE expression cassette | [^2^](#_ENREF_2) |
| pCFB3045 | NA | NA | 2µ, ori, AmpR, NatMXsyn, XI-3-gRNA | Template for PCR amplification of XI-3-gRNA expression cassette | [^4^](#_ENREF_4) |
| pEDJ366 | NA | NA | 2µ, ori, AmpR, NatMXsyn, URA3-gRNA | Introducing double strand break at URA3 locus(gRNA sequence TTAGCAGAATTGTCATGCAA); template for PCR amplification of URA3-gRNA expression cassette | In house |
| pTAJAK_71 | NA | NA | 2µ ori, AmpR, NatMXsyn, USER cassette | gRNA expression plasmid with NatMXsyn, used as backbone for multiple gRNA-donor constructs after enzyme digestion | [^4^](#_ENREF_4) |
| pCFB9967 | pTAJAK_71 | yEGFP-gRNA, XI-3-gRNA | 2µ, ori, AmpR, NatMXsyn, yEGFP-gRNA, XI-3-Grna | Introducing double strand break at yEGFP and XI-3 locus | USER cloning |
| pCFB9946 | pTAJAK_71 | ScARO1_E -gRNA, URA3-gRNA | 2µ, ori, AmpR, NatMXsyn, ScARO1_E -gRNA, URA3-gRNA | Introducing double strand break at ScARO1_E and URA3 locus | USER cloning |

Table S3 Synthesized DNA fragments carrying gRNA-donor that introduce mutations into *PYK1*

| Fragment | Sequence |
| --- | --- |
| frag_ PYK1A336S | cgcagtgaaagataaatgatcGTTGTCTGGTGAAACCGCCAgttttagagctagaaatagcaagttaaaataaggctagtccgttatcaacttgaaaaagtggcaccgagtcggtggtgctttttttgttttttatgtctACCCAAGACCAACCAGAGCTGAAGTTTCCGATGTCGGTAACGCTATCTTGGATGGTGCTGACTGTGTTATGctctcaggagagacaAGCaaaGGTAACTACCCAATCAACGCCGTTACCACTATGGCTGAAACCGCTGTCATTGCTGAACAAGCTATCGCTTACTTCGCCTTACTAGTACGTTCT |
| frag_PYK1D147N | cgcagtgaaagataaatgatcAGAATCATCTACGTTGATGAgttttagagctagaaatagcaagttaaaataaggctagtccgttatcaacttgaaaaagtggcaccgagtcggtggtgctttttttgttttttatgtctCGCTAAGGCTTGTGACGACAAGATCATGTACGTTGACTACAAGAACATCACCAAGGTCATCTCCGCTGGTcgtattatatatgtagacaacGGTGTTTTGTCTTTCCAAGTTTTGGAAGTCGTTGACGACAAGACTTTGAAGGTCAAGGCTTTGAACGCCGGTAATCGCCTTACTAGTACGTTCT |

Table S4 Primers used in this study

| Primers | Sequence | Note | Purpose |
| --- | --- | --- | --- |
| 26124 | gaattagatggtgatgttaaGTTTTAGAGCTAGAAATAGCAAGTTAAAATAAGGCTA | Whole plasmid PCR of pQC003 with yEGFP gRNA as the overhang | Construction of pCFB9940 via Gibson assembly |
| 26125 | ttaacatcaccatctaattcGATCATTTATCTTTCACTGCGGAG |  |  |
|  |  |  |  |
| 26132 | TGAAACTGAATCCGCACAATGTTTTAGAGCTAGAAATAGCAAGTT | Whole plasmid PCR of pQC003 with ScARO1E gRNA as the overhang | Construction of pCFB9941 via Gibson assembly |
| 26133 | ATTGTGCGGATTCAGTTTCAGATCATTTATCTTTCACTGCGGAG |  |  |
|  |  |  |  |
| 26148 | ggtagaattgacgctggtttGTTTTAGAGCTAGAAATAGCAAGTTAAAATAAGGCTA | Whole plasmid PCR of pQC003 with BenM gRNA as the overhang | Construction of pCFB9944 via Gibson assembly |
|  |  |  |  |
| 26149 | aaaccagcgtcaattctaccGATCATTTATCTTTCACTGCGGAG |  |  |
|  |  |  |  |
| 26150 | AGTGCAGGUAAAACAATGTCCCCTTCTAAAATGAATGCTAC | Amplification of PCK1 ORF with user overhangs from the CENPK genome | Construction of pCFB9945 via USER cloning |
| 26151 | aCGTGCGAUTTACTCGAATTGAGGACCAGCGG |  |  |
| 26152 | ATCTGTCAUAAAACAATGTCGCAAAGAAAATTCGCCG | Amplification of PYC1 ORF with user overhangs from the CENPK genome |  |
| 26153 | aCACGCGAUTCATGCCTTAGTTTCAACAGGAACTTG |  |  |
| 26154 | ACCTGCACUTTGTTTTATATTTGTTG | Amplification of TEF1p-PGK1p with user overhangs from pCFB8337 |  |
| 26155 | ATGACAGAUTTGTAATTAAAACTTAG |  |  |
| 26156 | ATCGCGTGuCATTCATCCGCTCTAACCGAAAAG | Amplification of the pCFB8337 backbone with user overhangs |  |
| 26157 | ATCGCACGuGTAGATACGTTGTTGACACTTCTAAATAAGCG |  |  |
|  |  |  |  |
| 10525 | CGTGCGAUagggaacaaaagctggagct | Expression cassettes of yEGFP-gRNA, TPO2 gRNA-donor, and Aro1_E-gRNA with user overhangs from the pCFB9940, pCFB8926, and pCFB9941, respectively | Construction of double gRNA plasmids pCFB9946, pCFB9947 and pCFB9967 via USER cloning |
| 24597 | ACCTGCACUagtaatacgactcactatagggcgaat |  |  |
| 10526 | AGTGCAGGUagggaacaaaagctggagct | Expression cassettes of XI-3-gRNA, and URA3-gRNA with user overhangs from the pCFB3045, and pEDJ366, respectively |  |
| 10529 | CACGCGAUtaactaattacatgactcga |  |  |
|  |  |  |  |
| 22769 | gaggattttcgatggagcaggatg | Flanking region for homologous recombination | Construction of XI-2-PGK1p-ScARO4_K229L via overlapping PCR |
| 22770 | ttgtggaagttcatggcaaacgctc |  |  |
| 26126 | gagcgtttgccatgaacttccacaaggaagtaccttcaaagaatggggtc | Fragment of PGK1p-ScARO4_K229L-CYC1t expression cassette |  |
| 22772 | aatatctgaaagcgctagtcgtgtgttcgagcgtcccaaaaccttctcaa |  |  |
| 22773 | cacacgactagcgctttcagatatt | Flanking region for homologous recombination |  |
| 22774 | gtgggaagattccgctctacca |  |  |
|  |  |  |  |
| 22769 | gaggattttcgatggagcaggatg | Flanking region for homologous recombination | Construction of XI-2-ADH1t-EcaroG_L175D-TEF1p or XI-2-ADH1t-EcAroG_S180F-TEF1p via overlapping PCR |
| 22770 | ttgtggaagttcatggcaaacgctc |  |  |
| 26130 | gagcgtttgccatgaacttccacaaGAGCGACCTCATGCTATACCTG | Fragment of expression cassette ADH1t-EcaroG_L175D-TEF1p or ADH1t-EcAroG_S180F-TEF1pamplified from pCFB1075 or pCFB1076 |  |
| 26129 | aatatctgaaagcgctagtcgtgtgCACACACCATAGCTTCAAAATGTTTCT |  |  |
| 22773 | cacacgactagcgctttcagatatt | Flanking region for homologous recombination |  |
| 22774 | gtgggaagattccgctctacca |  |  |
|  |  |  |  |
| 22769 | gaggattttcgatggagcaggatg | Flanking region for homologous recombination | Construction of XI-2-PGK1p-ScARO1_ΔE-CYC1t-ADH1t-ScARO4_K229L-TEF1p via overlapping PCR |
| 22770 | ttgtggaagttcatggcaaacgctc |  |  |
| 26128 | ttgagaaggttttgggacgctcgaaGAGCGACCTCATGCTATACCTG | Fragment of expression cassette ADH1t-ScARO4_K229L-TEF1p amplified from pCFB9114 |  |
| 26129 | aatatctgaaagcgctagtcgtgtgCACACACCATAGCTTCAAAATGTTTCT |  |  |
| 26126 | gagcgtttgccatgaacttccacaaggaagtaccttcaaagaatggggtc | Fragment of expression cassette PGK1p-ScARO1_ΔE-CYC1t amplified from pCFB8808 |  |
| 26131 | ttcgagcgtcccaaaaccttctcaattctcaagcaaggttttcagtataatgttaca |  |  |
| 22773 | cacacgactagcgctttcagatatt | Flanking region for homologous recombination |  |
| 22774 | gtgggaagattccgctctacca |  |  |
|  |  |  |  |
| 22750 | gcggagaagtcgttgatagcatttc | Flanking region for homologous recombination | Construction of XI-5-AroY.B-Ciso via overlapping PCR |
| 22751 | tggtgcacggagtttatggcaca |  |  |
| 22752 | tgtgccataaactccgtgcaccagagcgacctcatgctatacctgag | Fragment of AroY.B-Ciso expression cassette |  |
| 22753 | gggtgtactatgaagcagccaatattctcaagcaaggttttcagtata |  |  |
| 22754 | tattggctgcttcatagtacaccc | Flanking region for homologous recombination |  |
| 22755 | gatcatagatccggcacttagaga |  |  |
|  |  |  |  |
| 22756 | gtatccggctgttccttcatagcc | Flanking region for homologous recombination | Construction of XII-4-AroY.B-Ciso via overlapping PCR |
| 22757 | tgccatagtatgtgtgatggaaa |  |  |
| 22758 | tttccatcacacatactatggcagagcgacctcatgctatacctgag | Fragment of AroY.B-Ciso expression cassette |  |
| 22759 | cttttatttgactctaatggggaatttctcaagcaaggttttcagtata |  |  |
| 22760 | ATTCCCCATTAGAGTCAAATAAAAG | Flanking region for homologous recombination |  |
| 22761 | TTTCTGCTGTACCTGGATGGTC |  |  |
|  |  |  |  |
| 23617 | gtgatcattggcttaacgaaacggg | Flanking region XII-5UP for homologous recombination with overhang to TEF1p | Construction of XII-5-RKI1 via overlapping PCR |
| 25232 | AGTAAAAAAGGAGTAGAAACATTTTGAAGCTATtatagcggtctcctcccgtacc |  |  |
| 24748 | ATAGCTTCAAAATGTTTCTACTCCTTTTTTACT | TEF1p |  |
| 24749 | TTTGTAATTAAAACTTAGATTAGATTGCTATGCTTTCTTTCTAATG |  |  |
| 24749 | GAAAGCATAGCAATCTAATCTAAGTTTTAATTACAAAATGGCTGCCGGTGTCCCAAA | RKI1 ORF with overhang to TEF1p and CYC1t |  |
| 24797 | tccttttcggttagagcggatTCACTTTTCGGTAACTTCAACACTACCGTCAG |  |  |
| 24793 | atccgctctaaccgaaaagga | CYC1t with overhang to XII-5DW |  |
| 23620 | tgatgaacttgcttgctgtcaaactCTTCGAGCGTCCCAAAACCTT |  |  |
| 23621 | agtttgacagcaagcaagttcatca | Flanking region XII-5DW for homologous recombination |  |
| 23622 | gacctcttttgcctttcaaaaaagg |  |  |
|  |  |  |  |
| 23617 | gtgatcattggcttaacgaaacggg | Flanking region XII-5UP for homologous recombination with overhang to TEF1p | Construction of XII-5UP-TEF1p-RKI1-CYC1t via overlapping PCR |
| 25232 | AGTAAAAAAGGAGTAGAAACATTTTGAAGCTATtatagcggtctcctcccgtacc |  |  |
| 24748 | ATAGCTTCAAAATGTTTCTACTCCTTTTTTACT | TEF1p |  |
| 24749 | TTTGTAATTAAAACTTAGATTAGATTGCTATGCTTTCTTTCTAATG |  |  |
| 24749 | GAAAGCATAGCAATCTAATCTAAGTTTTAATTACAAAATGGCTGCCGGTGTCCCAAA | RKI1 ORF with overhang to TEF1p and CYC1t |  |
| 24797 | tccttttcggttagagcggatTCACTTTTCGGTAACTTCAACACTACCGTCAG |  |  |
| 24793 | atccgctctaaccgaaaagga | CYC1t |  |
| 24794 | cttcgagcgtcccaaaacctt |  |  |
|  |  |  |  |
| 24793 | atccgctctaaccgaaaagga | CYC1t | Construction of CYC1t-TDH3p-TKL1-ADH1t via overlapping PCR |
| 24794 | cttcgagcgtcccaaaacctt |  |  |
| 24796 | aaggttttgggacgctcgaagTCGAGTTTATCATTATCAATACTGCCATTTCA | TDH3p with overhang to CYC1t |  |
| 24754 | TTTGTTTGTTTATGTGTGTTTATTCGAAACTAAGTTCT |  |  |
| 24755 | AGTTTCGAATAAACACACATAAACAAACAAAATGACTCAATTCACTGACATTGATAAGC | TKL1 ORF with overhang to TDH3p and ADH1t |  |
| 24756 | ATAACTTATTTAATAATAAAAATCATAAATCATAAGAAATTCGCTTAGAAAGCTTTTTTCAAAGGAGAAATTAGCTTG |  |  |
| 24757 | GCGAATTTCTTATGATTTATGATTTTTATTATTAAATAAGTTAT | ADH1t |  |
| 24758 | GCATATCTACAATTGGGTGAAATGG |  |  |
|  |  |  |  |
| 24757 | GCGAATTTCTTATGATTTATGATTTTTATTATTAAATAAGTTAT | ADH1t | Construction of ADH1t-PGK1p-TAL1-FBA1t-XII-5DW via overlapping PCR |
| 24758 | GCATATCTACAATTGGGTGAAATGG |  |  |
| 24759 | CCATTTCACCCAATTGTAGATATGCACGCACAGATATTATAACATCTGCACA | PGK1p with overhang to ADH1t |  |
| 24760 | TTTGTTATATTTGTTGTAAAAAGTAGATAATTACTTCCTTGATGATCT |  |  |
| 24761 | AAGGAAGTAATTATCTACTTTTTACAACAAATATAACAAAATGTCTGAACCAGCTCAAAAGAAAC | TAL1 ORF with overhang to PGK1p and FBA1t |  |
| 24762 | CAGATTCAATACTCATTAAAAAACTATATCAATTAATTTGAATTAACTTAAGCGGTAACTTTCTTTTCAATCAAGTC |  |  |
| 24763 | GTTAATTCAAATTAATTGATATAGTTTTTTAATGAGTATTGAATCTGT | FBA1t |  |
| 24764 | AGTAAGCTACTATGAAAGACTTTACAAAGAACT |  |  |
| 25234 | AGTTCTTTGTAAAGTCTTTCATAGTAGCTTACTagtttgacagcaagcaagttcatca | Flanking region XII-5DW for homologous recombination |  |
| 23622 | gacctcttttgcctttcaaaaaagg |  |  |
|  |  |  |  |
| 4446 | gttgagctctgtccttcatggac | Flanking region XII-1UP for homologous recombination with overhang to TEF1p | Construction of XII-1UP-TEF1p-ZWF1-CYC1t via overlapping PCR |
| 24772 | GTAAAAAAGGAGTAGAAACATTTTGAAGCTATgaaagaaccgaaccgatgcc |  |  |
| 24748 | ATAGCTTCAAAATGTTTCTACTCCTTTTTTACT | TEF1p |  |
| 24749 | TTTGTAATTAAAACTTAGATTAGATTGCTATGCTTTCTTTCTAATG |  |  |
| 24773 | GAAAGCATAGCAATCTAATCTAAGTTTTAATTACAAAATGAGTGAAGGCCCCGTCAAAT | ZWF1 ORF with overhang to TEF1p and CYC1t |  |
| 24795 | tccttttcggttagagcggatCTAATTATCCTTCGTATCTTCTGGCTTAGTCACG |  |  |
| 24793 | atccgctctaaccgaaaagga | CYC1t |  |
| 24794 | cttcgagcgtcccaaaacctt |  |  |
|  |  |  |  |
| 24793 | atccgctctaaccgaaaagga | CYC1t | Construction of CYC1t-TDH3p-SOL3-ADH1t via overlapping PCR |
| 24794 | cttcgagcgtcccaaaacctt |  |  |
| 24796 | aaggttttgggacgctcgaagTCGAGTTTATCATTATCAATACTGCCATTTCA | TDH3p with overhang to CYC1t |  |
| 24754 | TTTGTTTGTTTATGTGTGTTTATTCGAAACTAAGTTCT |  |  |
| 24775 | AGTTTCGAATAAACACACATAAACAAACAAAATGGTGACAGTCGGTGTGTTTT | SOL3TKL1 ORF with overhang to TDH3p and ADH1t |  |
| 24776 | ATAACTTATTTAATAATAAAAATCATAAATCATAAGAAATTCGCCTAAAAAGTTTTCGTTTGAACTTTTCCAAAAGCT |  |  |
| 24757 | GCGAATTTCTTATGATTTATGATTTTTATTATTAAATAAGTTAT | ADH1t |  |
| 24758 | GCATATCTACAATTGGGTGAAATGG |  |  |
|  |  |  |  |
| 24757 | GCGAATTTCTTATGATTTATGATTTTTATTATTAAATAAGTTAT | ADH1t | Construction of ADH1t-PGK1p-GND1-FBA1t-XII-1DW via overlapping PCR |
| 24758 | GCATATCTACAATTGGGTGAAATGG |  |  |
| 24759 | CCATTTCACCCAATTGTAGATATGCACGCACAGATATTATAACATCTGCACA | PGK1p with overhang to ADH1t |  |
| 24760 | TTTGTTATATTTGTTGTAAAAAGTAGATAATTACTTCCTTGATGATCT |  |  |
| 24777 | AAGGAAGTAATTATCTACTTTTTACAACAAATATAACAAAATGTCTGCTGATTTCGGTTTGATT | GND1 ORF with overhang to PGK1p and FBA1t |  |
| 24778 | CAGATTCAATACTCATTAAAAAACTATATCAATTAATTTGAATTAACTTAAGCTTGGTATGTAGAGGAAGAAACAT |  |  |
| 24763 | GTTAATTCAAATTAATTGATATAGTTTTTTAATGAGTATTGAATCTGT | FBA1t |  |
| 24764 | AGTAAGCTACTATGAAAGACTTTACAAAGAACT |  |  |
| 24787 | TTCTTTGTAAAGTCTTTCATAGTAGCTTACTtcagtttagtgctctgtctgagtg | Flanking region XII-1DW for homologous recombination |  |
| 24780 | caatcctcgcatttcagcttcc |  |  |
|  |  |  |  |
| 22777 | gatcatttatctttcactgcggaga | Backbone with overhang to frag_ PYK1A336S and frag_PYK1D147N | Construction of pCFB9942 and pCFB9943 via Gibson assembly |
| 22776 | TCGCCTTACTAGTACGTTCTattcgccctatagtgagtcgtatta |  |  |
|  |  |  |  |
| 26134 | TTAACCTTTGATGACTTAACTGAACAAACTG | Flanking region (ARO1_D region) for homologous recombination with overhang to CYC1t | Construction of ARO1_D-CYC1t-ADH1t-EcaroE-promoters-ARO1t via overlapping PCR |
| 27021 | cttccttttcggttagagcggatctaGGATTCAGTTTCAAATTTATCGAACTTGTGA |  |  |
| 23568 | atccgctctaaccgaaaaggaagg | CYC1t-ADH1t |  |
| 26131 | ttcgagcgtcccaaaaccttctcaattctcaagcaaggttttcagtataatgttaca |  |  |
| 27022 | ATAACTTATTTAATAATAAAAATCATAAATCATAAGAAATTCGCtcaCGCGGACAATTCCTCCT | EcaroE with overhang to ADH1t, amplified from *E. coli* genome |  |
| 27023 | atgGAAACCTATGCTGTTTTTGGTAAT |  |  |
| 27024 | TACCAAAAACAGCATAGGTTTCcatTTAAAATTTAGTCTTAGATATAGAATGTAATCTTATTCTTTGATGTG | DAK1p with overhang to EcaroE and ARO1t |  |
| 26139 | CATTGTAAAATATAAAAAAGGATAGAGGTACTATTGTTCAAGAACATTGATCGGTTTGTTGTT |  |  |
| 27025 | TACCAAAAACAGCATAGGTTTCcatGATGCTTTGATTTTGTAGATATGTAGTTAAATAATTTTC | ADH5p with overhang to EcaroE and ARO1t |  |
| 26145 | CATTGTAAAATATAAAAAAGGATAGAGGTACTATTGTACTATGTCATTTTGAAGTTGGTTAGAATT |  |  |
| 27026 | TACCAAAAACAGCATAGGTTTCcatTTCTGATAGATTCTTTTGTTTACTAAATTTAGCGGTTA | ARO4p with overhang to EcaroE and ARO1t |  |
| 26756 | CATTGTAAAATATAAAAAAGGATAGAGGTACTATTGTTGTTGCAAGATAACAATACTGGCAAAC |  |  |
| 27028 | TACCAAAAACAGCATAGGTTTCcatttgtaattaaaacttagattagattgctatgctttctttc | TEF1p with overhang to EcaroE and ARO1t |  |
| 26760 | CATTGTAAAATATAAAAAAGGATAGAGGTACTATTGTgcacacaccatagcttcaaaatg |  |  |
| 26140 | ACAATAGTACCTCTATCCTTTTTTATATTTTACAATG | Flanking region of ARO1t for homologous recombination |  |
| 26141 | GTCTATGTGGACTTCTCATATCCCAAG |  |  |
|  |  |  |  |
| 26134 | TTAACCTTTGATGACTTAACTGAACAAACTG | Flanking region (ARO1_D region) for homologous recombination with overhang to partial CYC1t | Construction of ARO1_D-ARO1t via overlapping PCR |
| 27021 | cttccttttcggttagagcggatctaGGATTCAGTTTCAAATTTATCGAACTTGTGA |  |  |
| 27029 | atccgctctaaccgaaaaggaagACAATAGTACCTCTATCCTTTTTTATATTTTACAATG | Flanking region of ARO1t with overhang to partial CYC1t, for homologous recombination |  |
| 26141 | GTCTATGTGGACTTCTCATATCCCAAG |  |  |
|  |  |  |  |
| 23623 | AGTTACTTGCTCTATGCGTTTGCGC | Flanking region for homologous recombination | Construction of XI-3-ARO1_ΔE via overlapping PCR |
| 23624 | AATCAGACGCACGCTTGGCG |  |  |
| 26842 | tgtattgctggctcaatccacgtaaCTTCGAGCGTCCCAAAACCTT | Fragment PGK1p-ARO1_ΔE-CYC1t amplified from pCFB8808 |  |
| 26843 | cgccaagcgtgcgtctgattGGAAGTACCTTCAAAGAATGGGGTC |  |  |
| 23626 | TTACGTGGATTGAGCCAGCAATACA | Flanking region for homologous recombination |  |
| 23627 | TGAGAATCCGGACCAGCAGATAATG |  |  |
|  |  |  |  |
| 23822 | GACACATCTAACTGATTAGTTTTCCGTTTTAGGATATTGACGCCAAGCGTGCGTCTGATTGAGCGACCTCATGCTATACCTGAG | Fragment EcaroB-EcaroD amplified from p01955 with 60 bp overhangs containing homologous regions | Construction of XI-3-EcaroB-EcaroD via PCR |
| 23823 | ACTAACATCATGTACAAAACAGTTTAATAATGATCTGTATTGCTGGCTCAATCCACGTAACTTCGAGCGTCCCAAAACCTTCTC |  |  |
|  |  |  |  |
| 27054 | CGGTAATCTCCGAGCAGAAGGA | Flanking region (URA3-UP) with overhang to KlURA3 expressiong cassette, for homologous recombination | Construction of URA3_UP-KlURA3-URA3_DW via overlapping PCR |
| 27055 | tcctcgatagaacctaaataaaacgagctGGTTCCTTTGTTACTTCTTCCGCC |  |  |
| 24712 | agctcgttttatttaggttctatcgagga | KlURA3 expressiong cassette |  |
| 24713 | gatcccaatacaacagatcacgtga |  |  |
| 27056 | tcacgtgatctgttgtattgggatcGGTACTGTTGACATTGCGAAGAGC | Flanking region (URA3-DW) with overhang to KlURA3 expressiong cassette, for homologous recombination |  |
| 27057 | TGGTTCTGGCGAGGTATTGGATA |  |  |

References

1. K. D. Entian and P. Kotter, *Yeast Gene Analysis, Second Edition*, 2007, **36**, 629-666.

2. G. Wang, S. Ozmerih, R. Guerreiro, A. C. Meireles, A. Carolas, N. Milne, M. K. Jensen, B. S. Ferreira and I. Borodina, *ACS Synth Biol*, 2020, DOI: 10.1021/acssynbio.9b00477.

3. Q. L. Liu, T. Yu, X. W. Li, Y. Chen, K. Campbell, J. Nielsen and Y. Chen, *Nature Communications*, 2019, **10**.

4. M. M. Jessop-Fabre, T. Jakociunas, V. Stovicek, Z. Dai, M. K. Jensen, J. D. Keasling and I. Borodina, *Biotechnol J*, 2016, **11**, 1110-1117.

5. S. A. van der Hoek, B. Darbani, K. E. Zugaj, B. K. Prabhala, M. B. Biron, M. Randelovic, J. B. Medina, D. B. Kell and I. Borodina, *Front Bioeng Biotechnol*, 2019, **7**, 262.

6. M. L. Skjoedt, T. Snoek, K. R. Kildegaard, D. Arsovska, M. Eichenberger, T. J. Goedecke, A. S. Rajkumar, J. Zhang, M. Kristensen, B. J. Lehka, S. Siedler, I. Borodina, M. K. Jensen and J. D. Keasling, *Nat. Chem. Biol.*, 2016, **12**, 951-958.

7. A. Rodriguez, K. R. Kildegaard, M. Li, I. Borodina and J. Nielsen, *Metab. Eng.*, 2015, **31**, 181-188.

8. M. Babaei, G. M. Borja Zamfir, X. Chen, H. B. Christensen, M. Kristensen, J. Nielsen and I. Borodina, *ACS Synth Biol*, 2020, **9**, 1978-1988.
